# Myokinetic Stretching Exercise Versus Post-Isometric Relaxation Combined with Traction in Patients with Cervical Radiculopathy—A Randomized Clinical Trial

**DOI:** 10.3390/life15050721

**Published:** 2025-04-29

**Authors:** Fatima Saleem, Maryam Arshad, Sahreen Anwar, Elena Adelina Panaet, Dragoș Ioan Tohănean, Cristina-Ioana Alexe, Dan Iulian Alexe

**Affiliations:** 1 Department of Physical Therapy, Riphah International University, Faisalabad 38000, Pakistan; stepfatimahsaleem@gmail.com (F.S.); maryamarshad4201@gmail.com (M.A.); 2 University Institute of Physical Therapy, University of Lahore, Lahore 54590, Pakistan; sahreen.anwar@uipt.uol.edu.pk; 3 Department of Physical and Occupational Therapy, “Vasile Alecsandri” University of Bacău, 600115 Bacău, Romania; alexedaniulian@ub.ro; 4 Faculty of Physical Education and Mountain Sports, Transilvania University of Brașov, 500036 Brașov, Romania; dragos.tohanean@unitbv.ro; 5 Department of Physical Education and Sports Performance, “Vasile Alecsandri” University of Bacău, 600115 Bacău, Romania

**Keywords:** cervical radiculopathy, cervical traction, stretching technique

## Abstract

Background: Cervical radiculopathy is one of the frequent musculoskeletal problems prevalent in the general population, characterized by neck pain radiating to the upper limb. This study investigated the effects of the myokinetic stretching technique versus post-isometric relaxation (PIR) exercises with mechanical traction in patients with cervical radiculopathy. Methods: A single-blinded, randomized clinical trial was conducted from March 2023 to June 2023. Sixty-six patients with cervical radiculopathy were randomly assigned to two groups: Group A (n = 33) received myokinetic stretching exercises and Group B (n = 33) received isometric relaxation exercises, while mechanical cervical traction was applied to both groups as the baseline treatment. The treatment frequency was two sessions per week for eight weeks. Outcome measures were pain, range of motion, and neck disability measured through the Numerical Pain Rating Scale, a goniometer, and the Urdu version of the Neck Disability Index. Assessments were performed at the baseline and the 4th and 8th weeks of the treatment. Results: The between-group analysis showed a non-significant difference (*p* > 0.05). The within-group analysis showed (*p* < 0.001) significant improvement in both groups. Conclusion: This study concluded that the myokinetic stretching technique and post-isometric relaxation exercises combined with mechanical traction are effective in improving pain, range of motion, and neck disability in patients with cervical radiculopathy.

## 1. Introduction

Cervical radiculopathy is a major disabling condition characterized by neck pain radiating to one or both of the upper extremities [1]. It affects approximately 83 out of every 100,000 individuals, with the highest occurrence typically seen in people in their 40s globally, and 5–36% of all radiculopathies are cervical radiculopathy [2].

Advancements in technology and overuse of gadgets lead to aging, degenerative changes, and abnormal biomechanical forces, which ultimately cause compression resulting in cervical radiculopathy [3]. Arthritic changes, post-traumatic disc herniation, muscular spasm, and abnormal posture for an extended time contribute to foraminal stenosis, resulting in neurogenic symptoms throughout the root of the exiting nerve. Nonmechanical causes of foraminal narrowing are any spinal infection or tumor. Typical symptoms of cervical radiculopathy are neck pain, paresthesia, numbness, and brachialgia in a dermatomal and myotomal pattern [4]. Frequent headaches, scapular pain, and sensory and motor dysfunction in the upper extremities are often associated with cervical radiculopathy [5].

Repeated unidirectional movements due to work-related demands or workstation obligations narrow the space available for the nerve root exit, resulting in impingement and symptom exacerbation [6]. This results in muscle spasm, hypomobility, and a muscular imbalance as muscles on the affected side shorten and contralateral muscles are stretched. The major muscles supporting the cervical spine tend to be affected first, for example, upper fibers of the trapezius, scalene, and levator scapulae are weakened, and there is joint stiffness, capsule tightness, and postural defects that may then affect the rest of the body’s movement [7].

A variety of treatments are available for cervical radiculopathy, from conservative management to surgical approaches [8]. Physical therapy is an important intervention, which is relatively safe and effective in the management of cervical radiculopathy [9]. Physical therapy treatment is usually a combination of electrotherapy, exercise therapy, and manual therapy. Mechanical traction through an electric tractiser is one of the recommended treatment regimens to relieve compression from the neural structures in patients suffering from cervical radiculopathy [10]. The aim of mechanical traction is the effective separation of the affected segments without further damage to the soft tissues. Cervical traction with the proper angle and force provides longitudinal forces to skeletal and nonskeletal structures, thus improving the intraforaminal space and relaxation to the adjacent structures. According to an experimental study on adults with mechanical neck disorders, mechanical traction, either alone or in combination with other treatments, improved pain and disability. There was also an enhancement in patient satisfaction and global perceived effect [11]. It was suggested that the use of mechanical traction to reduce intervertebral pressure once or twice a week for 3 months is beneficial in the long term and avoids the risks of surgery. Myokinetic stretching technique (MST) is a form of myofascial release that involves active or passive stretching with movement until a desirable release from the taut band is achieved.

In a recent study, Upneja et al. compared the effects of myofascial release in different stretch positions for three different muscles in the body and concluded that myofascial release in the stretch position is more effective in lengthening the muscle [12]. PIR is one of the “muscle energy procedures,” which directly induces muscle relaxation and indirectly mobilizes the joints. The initial step is to take up the slack by placing the muscle in a stretched position, as described by Lewit. The stretch is continued to the point where the first slight resistance (or “barrier”) is felt. The overall goal of PIR treatment is to reduce muscle tension and relieve the resultant pain and dysfunction by restoring the full stretch length of the muscle [13]. In a recent study by Dudoniene et al., it was concluded that post-isometric relaxation is superior to static stretching in reducing neck pain and improving the range of motion in patients with non-specific neck pain [14].

The use of cervical traction combined with soft tissue techniques is being employed to relieve intense pain and disability. This study examined two interventions: myokinetic stretching exercises and post-isometric relaxation exercises, in conjunction with mechanical traction. The aim was to evaluate the combined impact of these interventions in reducing pain intensity, improving range of motion, and enhancing overall functional capacity in individuals suffering from musculoskeletal disorders.

## 2. Materials and Methods

### 2.1. Study Design

**Figure 1:**
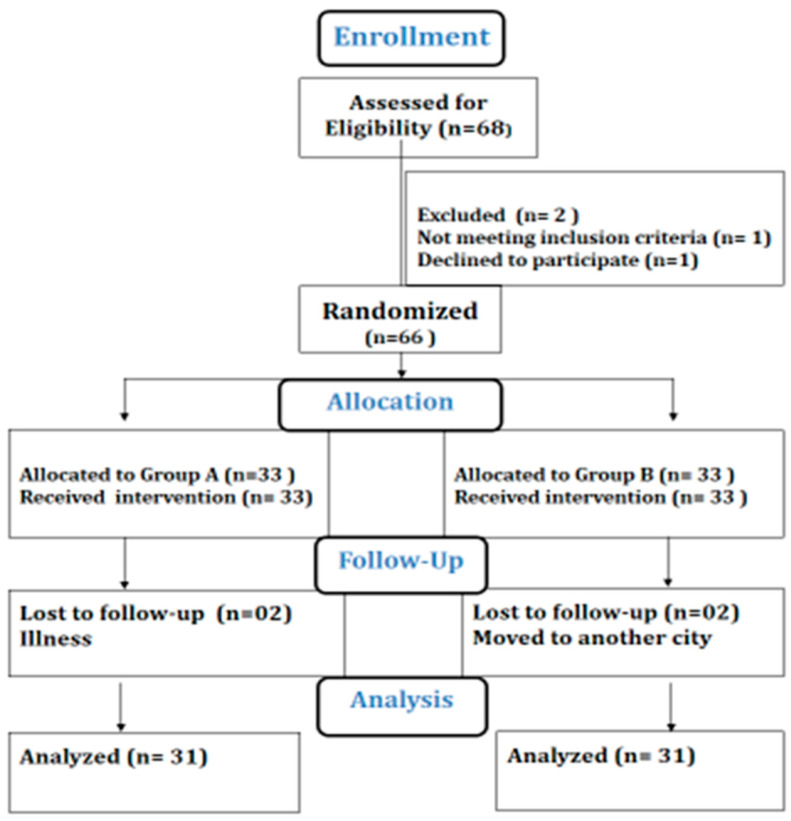
CONSORT chart.

This was a single-blinded, parallel, two-armed, randomized clinical trial conducted according to the consolidated standard of reporting trial (CONSORT) statement (Figure 1) [15]. According to the Declaration of Helsinki, all ethical considerations were assured, and ethical approval was obtained from the ethical review board of Riphah International University Faisalabad RCRAHS-REC/23/05 on 02.03.2023. The trial was prospectively registered in the clinical trials registry (NCT05812625). The study was conducted from March 2023 to June 2023 at the Physical Therapy department of Allied Hospital Faisalabad, Pakistan.

### 2.2. Inclusion and Exclusion Criteria

All the patients who fulfilled the inclusion criteria and expressed a willingness to participate in this study were included. The patients were briefed about the study, written informed consent was taken from the patients, and they were allowed to withdraw at any time. Male and female patients between the ages of 30 and 60 years with a positive Spurling test result and radiating arm pain for more than 4 weeks were included in this study. Patients with a history of surgery to the cervical spine, a spinal tumor, vertebral fracture, rheumatoid arthritis, osteoporosis, or prolonged use of steroids were excluded from this study through a multistep screening process [16].

### 2.3. Sample Size Calculation

A sample size of 68 patients was calculated through the Epitool calculator using a 5% margin of error, 5% confidence interval alpha, and 80% power. By adding a 10% attrition rate, we obtained a sample size of 62. The sample size was estimated based on the mean and standard deviation of pain scores in a study by Peter Emery (13). Patients were recruited from Allied Hospital Faisalabad through a non-probability convenience sampling technique.

### 2.4. Randomization and Masking

Randomization was performed by computer-generated allocation software version 2, and patients were randomly divided into two groups: A and B. Group A received mechanical cervical traction with myokinetic stretching exercises, and Group B received mechanical cervical traction with post-isometric relaxation exercises. This study was assessor-blinded, and allocation was carried out through computer software, with an even allocation to both groups.

### 2.5. Details of Intervention

After a thorough briefing about the intervention, the patient was asked to lie in a supine position. A thermal hot pack was applied for 10 min in the cervical region, followed by isometric exercises of the neck in 10 repetitions with a hold of 10 s, in all four directions. Mechanical cervical traction was applied by the Chattanooga Group Triton Traction unit, SKU 4749-INT model. The straps of the traction machine were wrapped around the neck, and the patient was advised to carefully place their head in the traction sling and try to relax as much as possible. A traction pull equivalent to one-eighth of the patient’s body weight was set on the traction machine panel with an intermittent force pattern of 20 to 30 s ON and 10 to 15 s OFF, for 15 min.

### 2.6. Myokinetic Stretching Technique (Group A)

The patient was placed in a supine position on the plinth, and the head was placed at the edge of the plinth by the therapist. The therapist held the neck in their hands in the neutral position and gradually bent it laterally to achieve cervical lateral flexion to the opposite side, to stretch the trapezius muscle. This maneuver was followed by myofascial release, by applying sustained finger pressure for 5–10 s on the involved trapezius. A gentle myofascial stretching force was applied to take up the slack and sustained until a release occurred. The MST protocol comprised three sets of four stretches with a 5 s rest period between the stretches. Treatment was provided twice a week for 8 weeks (7).

### 2.7. Post-Isometric Relaxation Exercise (Group B)

Cervical paraspinal post-isometric relaxation was performed with the patient supine, while the therapist slowly lifted the patient’s head toward the ceiling. Once a comfortable stretch was felt, the patient was asked to push their head back (with approximately 10% of their strength), while the therapist resisted the movement, thus creating an isometric contraction. This position was held for 8–10 s. The patient asked to inhale deeply and, upon exhalation, then instructed to relax while the therapist lifted the patient’s head a little further towards the ceiling. The protocol comprised three sets of post-isometric relaxation with 5 s rest periods. Treatment was provided twice a week for 8 weeks (13). The treatment duration was 40 min, and it was conducted 3 times a week.

### 2.8. Data Collection Instruments and Tools

The outcome measures were pain, range of motion, and neck disability measured through the Numerical Pain Rating Scale (NPRS), a goniometer, and the Neck Disability Index (NDI) in Urdu, respectively. The Numeric Pain Rating Scale (NPRS) was designated as the primary outcome measure in our study, whereas the secondary outcome measures were the Neck Disability Index (NDI), used to assess functional impairment related to neck pain, and range of motion (ROM), measured to determine any improvements in cervical mobility.

The Numeric Pain Rating Scale (NPRS) is a scale of 0 to 10 points, with 0 representing “no pain” and 10 representing “severe pain”. The NPRS is a valid and reliable instrument for measuring pain severity (15). The range of motion of the neck was measured by a universal goniometer, an instrument that either measures an angle or allows an object to be rotated to a precise angular position (16). The neck disability was measured through the Urdu version of the Neck Disability Index. The NDI is a 10-item questionnaire with a reasonable length and ease of use, which was developed to determine how neck disability affects daily tasks like personal hygiene, weight lifting, reading, headaches, concentration, work, driving, and sleeping. For a total score of 50, these 10 items can be valued up to 5 points per domain (17). Data were collected at baseline and in the 4th and 8th weeks of the treatment. The treatment duration was 40 min, and it was applied 3 times a week.

### 2.9. Statistical Analysis

Data were analyzed using the Statistical Package for Social Sciences (SPSS) version 26. Descriptive statistics, including the mean and standard deviation, were used to describe the demographics of patients. The normality of data was tested by the Kolmogorov–Smirnov test since data were not normally distributed. The non-parametric (Mann–Whitney U) test was used for between-group comparison, while the Friedman test was applied for within-group comparison at different time points (baseline, 4th and 8th weeks), and Bonferroni adjustment was used for pairwise comparison. A *p*-value < 0.05 was considered statistically significant for all analyzed data. All results were analyzed at a 95% confidence interval.

## 3. Results

During the eight weeks of the intervention program, 68 patients with cervical radiculopathy completed this study; the numbers of female and male participants were 16 (48.49%) and 17 (51.51%) in group A and 15 (45.45%) and 18 (54.54%) in group B, respectively. There were no significant differences between groups in the mean age, BMI (Body Mass Index), pain, or NDI. The average age, height, weight, and Body Mass Index of participants are given in Table 1.

The between-group analysis for the Numerical Pain Rating Scale and Neck Disability Index showed non-significant differences between both groups after the 8 weeks of the intervention. The mean rank values for the NPRS at baseline were (33.64) and (33.36) in the myokinetic stretching group and PIR group, respectively. In the 4th week of the intervention, the mean rank values were (32.15) and (33.0) and, in the 8th week of the intervention, the mean rank values were (30.24) and (32.76) in the MS group and PIR group, respectively. The *p*-value in between-group analyses in the 8th week was non-significant (0.561). For the Neck Disability Index, the mean rank values for the MS group and PIR group at baseline were (33.79) and (31.13), in the 4th week were (35.11) and (27.89), and in the 8th week were (35.37) and (27.63), respectively. The *p*-value in the 8th week was (0.089), showing a non-significant difference between the two groups (Table 2).

The between-group analysis for the cervical range of motion showed non-significant differences between both groups after the 8 weeks of the intervention. The mean rank values for cervical range of motion are presented in Table 3. The *p*-values for between-group analysis of cervical flexion and extension range of motion were non-significant at (0.242) and (0.098), while the *p*-values for right- and left-sided cervical rotation and cervical right- and left-sided lateral flexion, at (0.139), (0.072), (0.138), and (0.003), were also non-significant (Table 3).

Friedman’s test was used for within-group differences; the results of cervical flexion, extension, right rotation, left rotation, right lateral flexion, and left lateral flexion showed a significant improvement in both groups, with a *p*-value < 0.005 (Table 4).

## 4. Discussion

This randomized clinical trial investigated the effects of the myokinetic stretching technique and the post-isometric release exercise with traction in patients with cervical radiculopathy (CR). The results revealed that both methods effectively improve pain, range of motion, and disability in cervical radiculopathy.

The findings of this study are consistent with a study by Ghulam et al. where the post-isometric release technique in combination with cervical mobilization was found to be effective in alleviating pain and improving the range of motion and function in neck pain caused by a myofascial trigger point [17]. This two-arm, parallel, randomized clinical trial revealed that the post-isometric relaxation technique produced a significant improvement in both groups, with a *p*-value less than 0.05. Muscle spasms and trigger point formation are common problems in cervical radiculopathy, as degeneration not only triggers neural symptoms but also impacts the surrounding cervical spine muscles. Post-isometric release helps address muscle inhibition while promoting relaxation.

Hungud et al. compared the effects of myokinetic stretching exercises with cervical traction versus spinal mobilization with arm movement in thirty-two subjects with cervical radiculopathy [18]. According to the study’s findings, the myokinetic stretching technique (MST) and spinal mobilization with arm movement (SMWAM) are equally effective in reducing pain, enhancing range of motion (ROM), and reducing functional impairment in people with cervical radiculopathy. The myokinetic stretching group showed significant improvement from baseline measurements to the 6th day of the intervention. The major limitation of this study was the treatment duration, as one week is insufficient to predict any significant clinical change. In this study, we devised an 8-week intervention program, and assessments were performed at two time points, i.e., 4th week and the 8th week.

An improvement in neck pain and neurological symptoms caused by vertebral compression was also reported by Abd Elshafy et al. in a comparative study on intermittent and continuous cervical traction in mechanical neck pain [19]. The duration of the study was four weeks, and 12 sessions of cervical traction were provided to the patients with chronic neck pain. The study concluded that intermittent cervical traction is effective in reducing pain and mobility, but it had non-significant effects on functional disability. This might be because traction improved the symptoms of compression on neural structures, but the soft tissues caused disability and a hindrance in daily life activities. In our study, the combination of cervical traction and myokinetic stretching addressed the neurogenic and myogenic symptoms simultaneously, leading to improvements in pain, range, and disability.

In a study by Savva et al., it was concluded that the combination of cervical traction and neuromobilization produced an improvement in pain, function, and disability. That was a randomized clinical trial conducted on 66 patients with cervical radiculopathy between 50 and 60 years of age. The results showed an improvement in pain, function, and disability when cervical traction and neural mobilization were combined. These results advocate for the findings in our study, where traction was implemented via two manual therapy approaches for subjects between the ages of 30 and 60 years, including males and females, with a focus on manual therapy with mechanical traction. The outcomes of the current study were better for traction, where the *p*-value was 0.93 for both the post-isometric relaxation exercises and the myokinetic stretching exercises. There was a non-significant difference between groups. Patients with cervical radiculopathy experienced a reduction in pain and improved range of motion and functional status [11].

In 2022, Zainab Khalid et al. conducted a randomized clinical trial on 60 patients with non-specific neck pain to explore the effect of post-isometric relaxation compared to myofascial release therapy. According to the results, patients with non-specific neck pain can benefit from post-isometric relaxation, with significant improvements in pain, disability, cervical ROM, and quality of life compared with myofascial release therapy [20]. The results of this study support our findings as both techniques were beneficial in improving pain, range of motion, and disability in patients with cervical radiculopathy.

M. Rezwan et al. conducted an experimental study in 2022 on 30 cervical radiculopathy patients treated by cervical traction and neck isometric exercises. In this study, both genders were sampled with ages from 30 to 50 years. The experimental and control groups (n = 15) received mechanical cervical traction along with neck exercises, whereas the control group received only manual cervical traction once a day for four weeks. Manual traction was more effective than mechanical cervical traction. In the current research using traction, we applied two manual therapy techniques on patients between the ages of 30 and 60 years. Myokinetic stretching of the trapezius muscle was conducted in group A, and post-isometric relaxation in group B. When compared to group A (myokinetic stretching), group B (post-isometric relaxation) performed better over the 8-week treatment period. The results indicated that subjects with cervical radiculopathy have improved levels of discomfort, range of motion, and functional status [21].

A study by Y Kim et al. (2024) examined the effects of myofascial release and cervical traction in patients with chronic neck pain. It concluded that the combination of myofascial release with cervical traction is more effective in reducing pain and disability [22]. This study supports our findings as the application of cervical traction with myokinetic stretching and post-isometric relaxation resulted in improved pain, range of motion, and neck disability in patients with cervical radiculopathy.

Understanding the distinction between true cervical radiculopathy and radiating pain is essential for clinicians in accurately diagnosing and tailoring treatment approaches. True cervical radiculopathy is characterized by neurological symptoms resulting from nerve root compression, often presenting as pain, numbness, or weakness in specific dermatomes. In contrast, radiating pain may occur without neurological involvement, typically stemming from muscular or ligamentous sources. Clinicians should prioritize specific interventions for patients with confirmed cervical radiculopathy, such as targeted exercises and mechanical traction, which address the underlying nerve root irritation. For those experiencing radiating pain without true radiculopathy, a different treatment focus may be warranted, emphasizing pain management techniques and rehabilitation strategies to alleviate discomfort and improve functional outcomes.

When relating our findings to the existing literature, it becomes evident that accurately differentiating between these conditions can significantly enhance clinical practice. Previous studies have underscored the importance of precise diagnosis in guiding effective treatment strategies. By integrating our insights into clinical practice, healthcare providers can better tailor interventions to meet the specific needs of their patients, ultimately improving outcomes for individuals with cervical radiculopathy and related conditions. This nuanced understanding not only aids in effective treatment planning but also contributes to more informed patient education and engagement in their recovery process.

### Limitations

One limitation of this research is the possibility that some individuals may not have had true cervical radiculopathy, which could affect the generalizability of the findings and the interpretation of the effectiveness of the interventions. The major limitation of this study was the use of intermittent cervical traction, while the effect of continuous traction was not evaluated. There was a lack of a control group or placebo treatment because no radiculopathy patient could receive “no treatment” given that they had pain and disability. The study duration was limited to six weeks and there was no follow-up, so it is hard to understand how long the treatment effects of cervical traction and a combined soft tissue approach can benefit patients with cervical radiculopathy.

## 5. Conclusions

The current study concluded that the myokinetic stretching technique and post-isometric relaxation combined with traction are effective in reducing pain and improving the range of motion and neck disability in patients with cervical radiculopathy.

## Figures and Tables

**Table 1:** Demographic data.

	Mean ± SD (n = 33) Group A (MS)	Mean ± SD (n = 33) Group B (PIR)
Age (years)	42.16 ± 8.65	40.12 ± 7.61
Height (cm)	164.03 ± 13.37	166.02 ± 12.21
Weight (kg)	62.89 ± 11.09	61.10 ± 10.12
BMI	21.23 ± 4.22	20.13 ± 3.11
Gender	Frequency (%)	Frequency (%)
Male	16 (48.49%)	15(45.45%)
Female	17 (51.51%)	18(54.54%)

cm = centimeter, Kg = kilogram, BMI = Body Mass Index, SD = standard deviation.

**Table 2:** Between-group differences in NPRS and NDI at the baseline and 4th and 8th weeks.

	Groups	N	Mean Rank	Standard Deviation	Mann–Whitney U	Z	*p*-Value
NPRS Baseline	A	33	33.64	1.10	540.000	−0.059	0.953
	B	33	33.36	1.08			
4th week	A	31	32.15	0.09	460.500	−0.293	0.770
	B	31	33.00	1.01			
8th week	A	31	30.24	1.12	441.500	−0.581	0.561
	B	31	32.76	1.23			
NDI Baseline	A	33	33.79	0.07	469.000	−0.573	0.567
	B	33	31.13	1.11			
4th week	A	31	35.11	1.08	368.500	1.583	0.114
	B	31	27.89	1.07			
8th week	A	31	35.37	1.08	360.500	1.699	0.089
	B	31	27.63	0.02			

NPRS = Numeric Pain Rating Scale, NDI = Neck Disability Index

**Table 3:** Between-group difference in CROM at the baseline and 4th and 8th weeks.

Outcomes	Group	N	Mean Ranks	Standard Deviation	Mann–Whitney U	Z	*p*-Value
Flex baseline	A	33	31.32	1.10	472.50	−0.940	0.347
	B	33	35.68	1.90			
4th week	A	31	27.97	2.17	371.00	−1.616	0.106
	B	31	35.03	2.11			
8th week	A	31	28.87	1.18	399.00	−1.171	0.242
	B	31	34.13	1.95			
Ext baseline	A	33	33.30	2.10	538.00	−0.086	0.932
	B	33	33.70	2.13			
4th week	A	31	28.42	1.18	385.00	−1.370	0.171
	B	31	34.58	1.20			
8th week	A	31	35.19	2.13	366.00	−1.656	0.098
	B	31	27.81	3.12			
Rt Rot baseline	A	33	32.44	1.23	509.50	−0.027	0.978
	B	33	34.56	1.29			
4th week	A	31	30.26	1.45	444.00	−0.721	0.471
	B	31	32.74	1.90			
8th week	A	31	28.55	1.68	389.50	−1.481	0.139
	B	31	34.45	2.13			
Lt Rot baseline	A	33	34.30	1.21	518.00	−0.346	0.730
	B	33	32.70	1.45			
4th week	A	31	33.34	2.20	423.50	−0.818	0.413
	B	31	29.66	2.90			
8th week	A	31	35.56	1.28	354.50	−1.800	0.072
	B	31	27.44	1.54			
Rt lat Flex baseline	A	33	31.14	1.69	466.50	−1.128	0.259
	B	33	32.86	2.34			
4th week	A	31	32.69	2.19	443.50	−0.540	0.589
	B	31	33.31	1.18			
8th week	A	31	34.23	0.09	379.00	−1.484	0.138
	B	31	34.77	1.26			
Lt lat Flex baseline	A	33	30.04	1.32	466.50	−1.128	0.259
	B	33	34.09	1.48			
4th week	A	31	32.98	1.26	371.50	−1.663	0.096
	B	31	33.02	1.34			
8th week	A	31	35.85	1.21	274.50	−2.947	0.003
	B	31	36.15	1.32			

CROM, cervical range of motion Rt = right, Lt = left, Lat = lateral, Rot = rotation, Flex = flexion, Ext = extension, Rot = rotation.

**Table 4:** Within-group differences in the Numeric Pain Rating Scale and cervical range of motion.

Assessments		Group A	Group B
	NPRS Baseline	2.97	2.98
	NPRS Week 4	2.03	1.98
	NPRS Week 8	1.00	1.03
Chi-Square		61.049	59.528
*p*-Value		<0.001	<0.001
	NDI Baseline	2.44	2.50
	NDI Week 4	2.44	2.27
	NDI Week 8	1.13	1.23
Chi-Square		47.032	37.411
*p*-Value		<0.001	<0.001
	Flex Baseline	1.26	1.05
	Flex Week 4	1.81	1.97
	Flex Week 8	2.94	2.98
Chi-Square		48.867	58.585
*p*-Value		<0.001	<0.001
	Ext Baseline	1.18	1.21
	Ext Week 4	1.85	2.11
	Ext Week 8	2.97	2.68
Chi-Square		55.593	34.260
*p*-Value		<0.001	<0.001
	Rt Rot Baseline	1.37	1.23
	Rt Rot Week 4	2.47	2.39
	Rt Rot Week 8	2.16	2.39
Chi-Square		26.761	38.400
*p*-Value		<0.001	<0.001
	LT Rot Baseline	1.03	1.35
	LT Rot Week 4	2.52	2.40
	LT Rot Week 8	2.45	2.24
Chi-Square		46.621	19.919
*p*-Value		<0.001	<0.001
	Rt Lat Flex Baseline	1.06	1.13
	Rt Lat Flex Week 4	1.94	1.89
	Rt Lateral Flexion Week 8	3.00	2.98
Chi-Square		59.213	56.83
*p*-Value		<0.001	<0.001
	LT Lat Flex Baseline	1.02	1.00
	LT Lat Flexion Week 4	2.16	2.05
	LT Lateral Flexion Week 8	2.82	2.95
Chi-Square		53.966	59.626
*p*-Value		<0.001	<0.001

Rt = right, LT = left, Lat = lateral, Rot = rotation, Flex = flexion, Ext = extension, Rot = rotation.

## Data Availability

The data presented in this study are available on request from the corresponding author due to privacy and ethical restrictions.
